# Arthroscopic Imbrication of the Anterior Bundle of the Elbow Medial Collateral Ligament With a 70° Arthroscope (MI70 Procedure)

**DOI:** 10.1016/j.eats.2024.103417

**Published:** 2025-01-10

**Authors:** Paolo Arrigoni, Valerio Monteleone, Valeria Vismara, Simone Cassin, Elias Kaleb Rojero-Gil, Francesco Luceri, Aurelien Traverso, Carlo Zaolino, Pietro Simone Randelli

**Affiliations:** a Clinica Ortopedica, Azienda Socio Sanitaria Territoriale Centro Specialistico Ortopedico Traumatologico Gaetano Pini-CTO, Milan, Italy; b Scuola Di Specializzazione in Ortopedia e Traumatologia Università Degli Studi Di Milano, Milan, Italy; c Unidad Médica de Alta Especialidad Hospital de Traumatología y Ortopedia Manuel Ávila Camacho, Instituto Mexicano del Seguro Social, Puebla, México; d Service d’orthopedie et de traumatologie, Centre Hospitalier Universitaire Vaudois (CHUV), Lausanne, Switzerland; e Laboratory of Applied Biomechanics, Department of Biomedical Sciences for Health, Università Degli Studi Di Milano, Milan, Italy; f U.O.C. 1°Clinica Ortopedica, ASST Centro Specialistico Ortopedico Traumatologico Gaetano Pini-CTO, Milan, Italy; g Research Center for Adult and Pediatric Rheumatic Diseases (RECAP-RD), Department of Biomedical Sciences for Health, Università Degli Studi Di Milano, Milan, Italy

## Abstract

Most medial collateral injuries require very challenging therapeutic management, even for expert surgeons. After the failure of conservative treatment, the physician’s first choice should be a minimally invasive surgical technique. Reconstruction of the medial collateral ligament of the elbow with graft is the most described, demanding, and time-consuming procedure compared to plication of the ligament. This Technical Note aims to validate the results concerning a surgical technique, the MI70 procedure, elaborated to imbricate the anterior bundle of the medial collateral ligament and the anteromedial capsule through a suture anchor, through a 70° scope vision (MI70).

The medial collateral ligament (MCL) of the elbow is the primary static valgus stress stabilizer of the joint.^1^^,^^2^ It comprises an anterior band, a posterior band, and a transverse ligament.^1^ The anterior medial collateral ligament (aMCL) runs from the anteroinferior surface of the medial epicondyle to the medial aspect of the sublime tubercle, representing the primary restraint to valgus stress.^3^ Medial instability of the elbow can be traumatic^4^ or more frequently progressive.^5^ Most patients tolerate well-isolated aMCL insufficiency without consequences in daily activities, but others may develop symptomatic medial progressive instability, which manifests as medial proximal elbow pain and apprehension.^6^

The aMCL complete visualization is challenging during elbow arthroscopy.^7^ Less than half of the aMCL is visible with the standard 30° scope from the posterior portal; the use of a 70° scope positioned in the anterolateral portal could improve the procedure, allowing the surgeon to have a frontal view of the elbow joints.^8^ Several techniques for aMCL reconstruction based on the use of tendon grafts have been described.^9^ However, these grafts have different mechanical properties from the original ligament.

This Technical Note aims to clearly define the steps for a purely arthroscopic technique, the medial imbrication through a 70° scope vision (MI70).

## Surgical Technique

The surgery procedure is shown in Video 1; the main steps are described in the following section.

**Fig 1:**
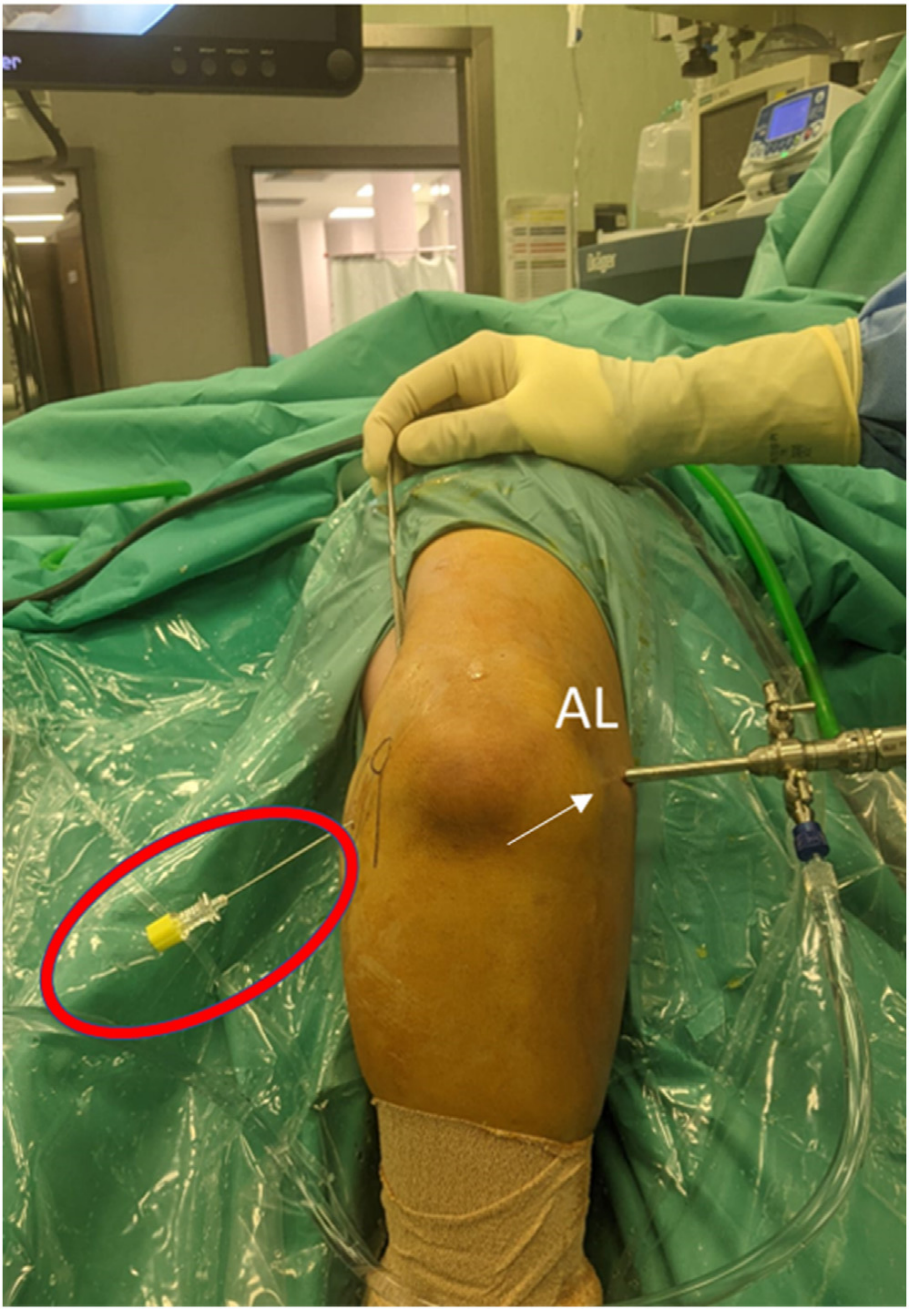
Right arm, frontal view, the arthroscope is placed through the anterolateral portal (white arrow), while a spinal needle (red circle) is inserted directed toward the intra-articular humeral emergence of the anterior bundle of the medial collateral ligament with an approximately 45° angle distal to proximal entering the skin in line with the medial epicondyle. This approach allows approaching the joint from a safe trajectory into a flexor-pronator mass. (AL, anterolateral.)

**Fig 2:**
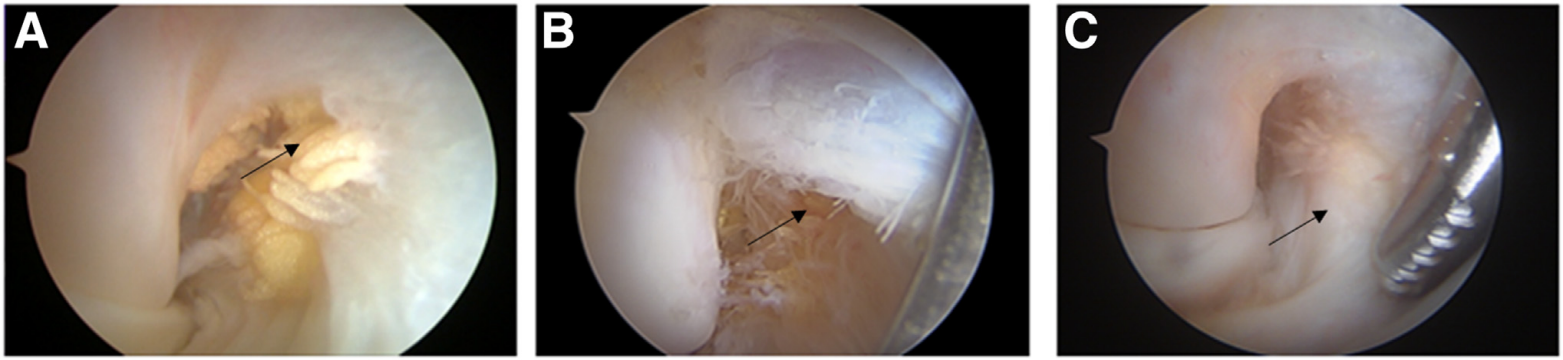
Vision of the right medial elbow gutter via the 70° scope through the anterolateral portal, showing traumatic anterior bundle of the medial collateral ligament (aMCL) tear (black arrow) (A), aMCL overstretch elongation (black arrow) (B), and healthy aMCL at humeral insertion (black arrow) (C).

The patient is set in a modified lateral decubitus position, with the operative arm at 100° of flexion of the shoulder and the use of a dedicated arm holder. The elbow is positioned at 90° of flexion, with the forearm hanging free to gravity with 20 to 30 cc of saline used to distend the elbow joint. The posterior compartment is assessed first with the elbow at 70° of flexion. After the first diagnostic phase looking from the anteromedial portal, the arthroscope is switched to the anterolateral portal, and a 70° scope is inserted (Fig 1). This approach allows a complete and frontal visualization of the medial gutter (Fig 2).

**Fig 3:**
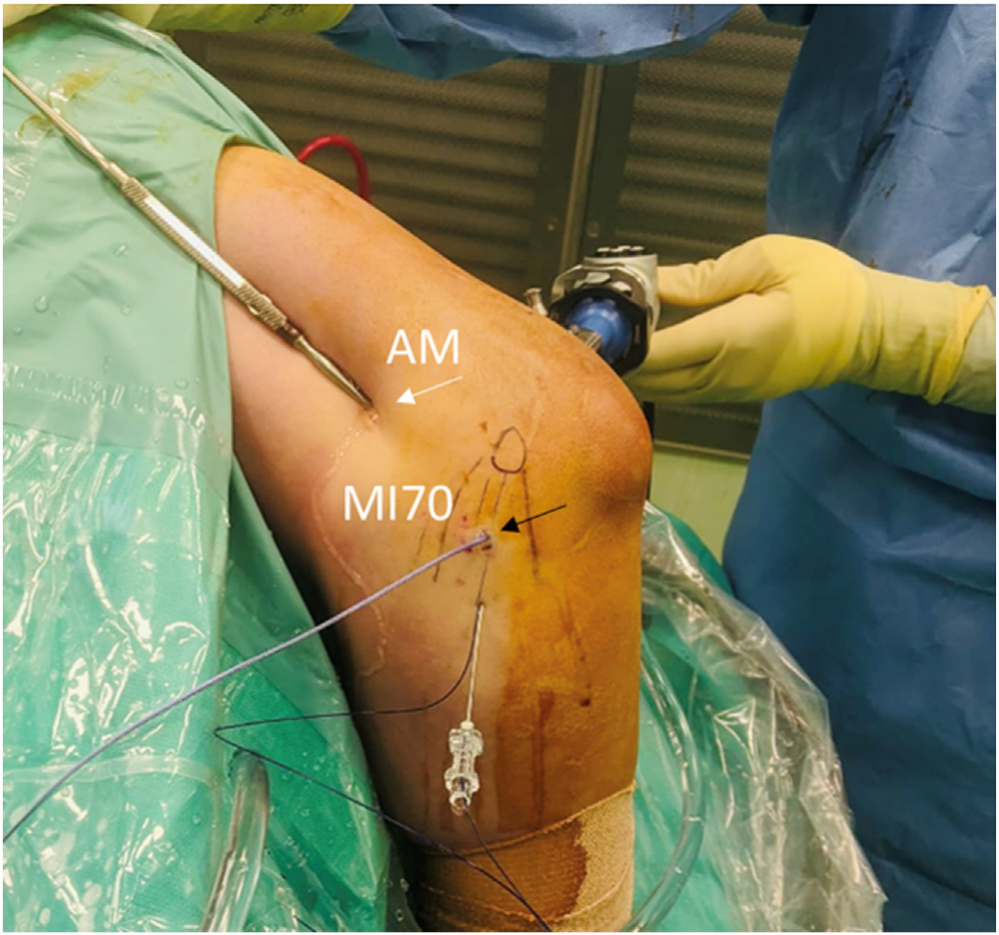
Lateral view of the medial side of the right elbow in lateral decubitus. Through the anteromedial portal (white arrow), a blunt retractor is held to facilitate capsular distention. A new portal (MI70, black arrow) is created following the needle direction and allows the introduction of a space drill and a bioabsorbable double-loaded suture anchor. (AM, anteromedial.)

Through the anteromedial portal, a retractor is positioned to facilitate capsular distention. Once ligamentous pathology is confirmed, either by a clear lesion or by the presence of synovitis, poor fiber quality, and obvious degeneration, a spinal needle (18 gauge) is inserted directed toward the intra-articular humeral emergence of the aMCL with an approximately 30° to 45° angle distal to proximal entering the skin in line with the medial epicondyle and central considering the flexor mass, as previously described.^8^ The needle enters 3 to 4 cm distal to the medial epicondyle and points toward the center of the medial gutter of the elbow. Once the position is confirmed, a dedicated portal (MI70 portal) is established following the needle direction. The portal is thereby created 3 to 4 cm distal to the medial epicondyle, which is in line with it and central, considering the flexor mass. This MI70 portal first introduces a drill guide (Depuy Mitek) pointing toward the center of the medial gutter and, second, a bioabsorbable double-loaded suture anchor (Fig 3).

**Fig 4:**
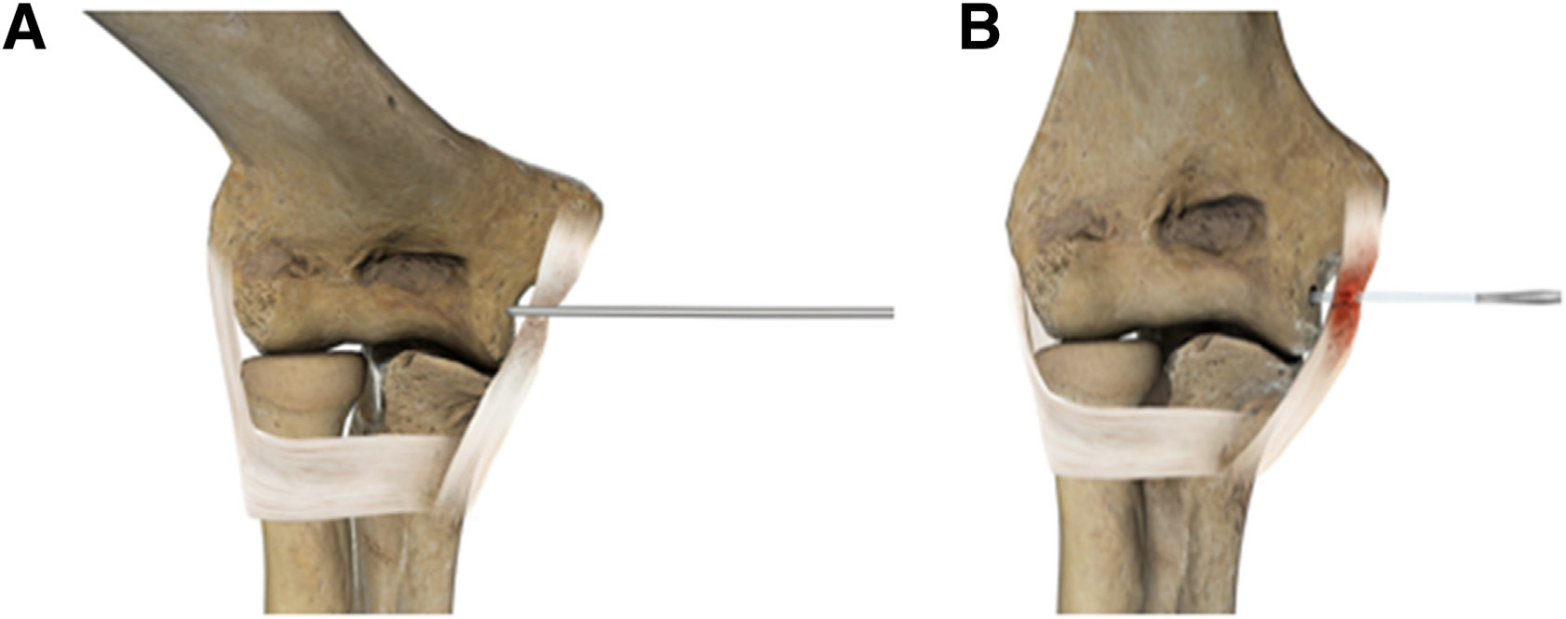
Anterior graphical representation of the right elbow drilling (A) and suture anchor placement (B), distal to the native anterior bundle of the medial collateral ligament insertion in the high anteromedial aspect of the medial gutter, parallel to the central axis of the trochlea.

The drill guide, always through the MI70 portal, together with the drill, is then directed at the bony curvature between the medial epicondyle and the outer part of the humeral trochlea, approximately 1 cm distal to the native aMCL insertion. Drilling is performed with an angle parallel or slightly distal to proximal to the central axis of the trochlea to avoid cortical thinning at the level of the olecranon fossa (Fig 4). Once the drilling is completed, a bioabsorbable double-loaded suture anchor is used (Lupine; Depuy Mitek).

**Fig 5:**
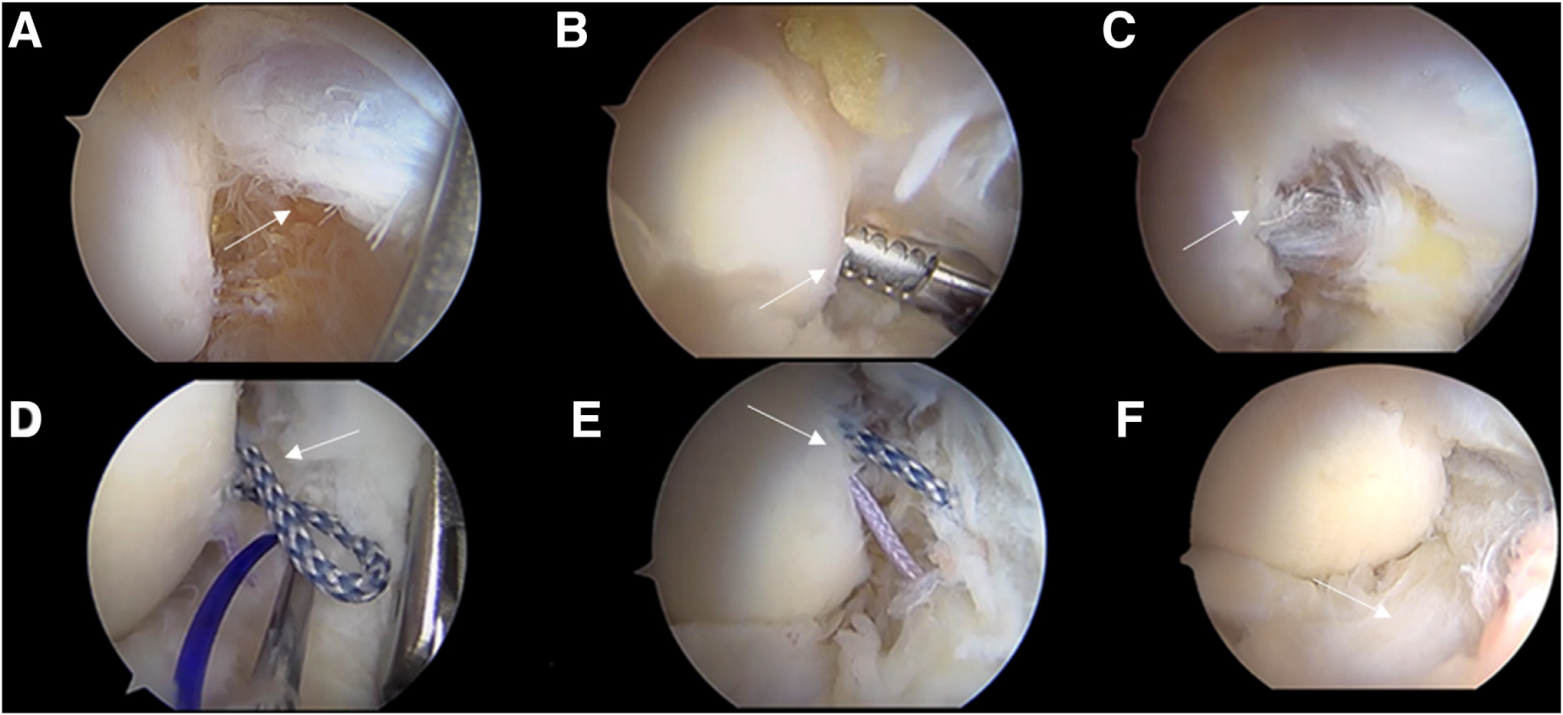
Vision from the anterolateral portal showing step-by-step arthroscopic anterior bundle of the medial collateral ligament (aMCL) plication. (A) Diagnostic evaluation of aMCL tear (white arrow). (B) Preparation of anchor placement on the medial trochlea (white arrow). (C) Spade drill introduction (white arrow). (D) Sutures shuttled through the PDS suture within the aMCL (white arrow). (E) Sutures passed through the aMCL (white arrow). (F) aMCL repair after suture tightening.

A PDS wire is then delivered through a needle inserted again in line with the MI70 portal, 1 to 1.5 cm to it and penetrating approximately 1 to 1.5 cm distal to the anchor placement, entering with a posterior-to-anterior direction and in line with and passing through the aMCL. A suture retriever is inserted via the MI70 portal to retrieve a suture from the anchor into the PDS looped back over the needle. The retrieving phase can sometimes be facilitated using a 30° scope if the loop happens to be positioned quite central into the anterior elbow chamber (Fig 5). The sutures passed through the aMCL are then retrieved subcutaneously via the MI70 portal, and a standard low-profile sliding knot is performed.

## Discussion

We describe an arthroscopic technique for MCL plication. Pearls and pitfalls are summarized in Table 1. The aMCL reconstruction with graft is nowadays the most described and employed surgical treatment for medial ligament injuries.^10^ Nevertheless, aMCL plication with a suture anchor appeared to be a valid alternative in patients with partial ligament damage. This technique could provide an anatomic and less invasive approach compared to graft reconstruction.

**Table 1:** Pearls and Pitfalls of the MI70 Procedure

MI70 Procedure Pearls	MI70 Procedure Pitfalls
This all-arthroscopic technique allows a dynamic evaluation of the intra-articular and ligamentous elbow structures before and after retensioning of the aMCL.	Difficult procedure that requires expert arthroscopic elbow surgeon.
The use of a 70° scope allows good visualization of the medial elbow compartment and allows good anchor placement.	The use of a 70° scope is highly recommended for good visualization of anchor placement.
	Incorrect and tangent placement of the anchor, with respect to the medial trochlea, due to poor visualization may result in anchor pull-out.

MI70 Procedure Advantages	MI70 Procedure Disadvantages

Allows minimally invasive ligament retensioning.	If the aMCL is severely injured, reconstruction with graft is the treatment of choice.
Early procedure to avoid the complete rupture of the aMCL and the development of other intra-articular problems.	The surgeon must be familiar with the use of a 70° scope.
Can provide a reduction in procedure-related morbidity and early return to daily life activities and sports compared to more invasive procedures.	

aMCL, anterior bundle of the medial collateral ligament.

The all-arthroscopic technique can provide a reduction in procedure-related morbidity,^11^ an opportunity to visualize and treat all elbow compartments, and the capability of performing a dynamic evaluation of the intra-articular and ligamentous structures before and after retensioning.^12^

Nowadays, there is no consensus regarding the ideal surgical treatment of aMCL injury. Most authors advised a direct repair of the ligament’s mid-substance rupture after an acute injury.^13^^,^^14^ In the same way, most bony ligamentous avulsions are openly reduced with screws.^14^ Reconstruction of the MCL with tendon graft is suggested as the treatment of choice by different authors^15^, ^16^, ^17^ in case of complete aMCL rupture, except in patients with very specific characteristics: acute traumatic event, normal quality of the ligament, and absence of ulnar nerve symptoms.

The aMCL imbrication with a suture anchor appears to be a valid alternative to reconstructions with grafts in the treatment of medial elbow pain in patients with partial ligament rupture or degeneration.^16^^,^^18^ This could be considered an early procedure to avoid the complete rupture of the aMCL and the development of other intra-articular problems, consequently sparing patients from more invasive techniques. Before surgery, it is extremely important to evaluate the patients through advanced imaging to grade the ligamentous lesion of the elbow.^19^^,^^20^ If the aMCL is severely injured, reconstruction with graft is the treatment of choice. Therefore, for now, the indications should be limited to those patients with medial elbow laxity who report pain and instability for a long period and are unresponsive to conservative treatments.^16^

The MI70 procedure is a technique that allows minimally invasive ligament retensioning. The aMCL plication with a suture anchor appeared to be a valid alternative to reconstructions with grafts in treating medial elbow ligament ruptures or elongations.

## Disclosures

All authors (P.A., V.M., V.V., S.C., E.K.R-G., F.L., A.T., C.Z., P.S.R.) declare that they have no known competing financial interests or personal relationships that could have appeared to influence the work reported in this paper.

## Supplementary Data

Video 1 The arthroscopic right elbow medial collateral ligament imbrication. A 70° scope in the anterolateral portal allows observation of the medial gutter, where the insertion of the medial collateral ligament is located. In some cases, the insertion site displays a distinct lesion. This technique is based on the creation of the MI70 portal. The portal is created at a distance of approximately 2 cm from the medial epicondyle, in alignment with this point. The needle is inserted at an angle of approximately 45° concerning the medial facet of the trochlea, and the procedure is conducted through the aforementioned portal with the aid of a shaper. The anchor must be inserted in alignment with the trans-epicondyle line. It is important to exercise caution as the view may be compromised by the frontal prospective. The anchor is mounted and inserted on the medial side of the medial trochlea; thus, it is not an atomic anchor. A transcutaneous suture technique is employed through an accessory. About the entry point, a suture is delivered with a PDS through a needle and retrieved through the MI70 portal using a classic technique. It is of the utmost importance that the needle enters posterior to anterior. It is typical for the passage to be repeated twice, thus creating 2 sutures and imbricating some fibers of the medial mass of the flexor tendons together with the ligament at the very end. This provides the final view.
